# Validation and Adaptation of the Nottingham Hip Fracture Score to Predict 30-Day and 1-Year Mortality Among Italian Older Adults Hospitalized Due to Hip Fractures

**DOI:** 10.3390/jcm15010310

**Published:** 2025-12-31

**Authors:** Valentina Gemo, Vittorio Bini, Ilaria G. Macchione, Lorenzo Lucchetta, Chiara Properzi, Federica Perini, Marta Baroni, Paola Montanari, Chiara Merenda, Fabio Gori, Barbara Bifarini, Enrico Iotti, Lorenzo Di Giacomo, Sabrina Ronzoni, Chiara Bendini, Paolo Pignedoli, Giuseppe Rinonapoli, Carmelinda Ruggiero

**Affiliations:** 1 Orthogeriatric and Geriatric Units, Gerontology and Geriatrics Section, Department of Medicine and Surgery, University of Perugia, S. Maria Misericordia Hospital, S. Andrea delle Fratte, 06132 Perugia, Italy; 2 Orthogeriatric and Geriatric Units, Degli Infermi Hospital, 48018 Faenza, Italy; 3 Orthopaedics and Traumatology Units, Department of Medicine and Surgery, University of Perugia, S. Maria Misericordia Hospital, S. Andrea delle Fratte, 06132 Perugia, Italy; 4 Orthogeriatrics, Neuromotor and Rehabilitation Department, Arcispedale S. Maria Nuova, 42121 Reggio Emilia, Italy; 5 Anaesthesia and Resuscitation, Department of Medicine and Surgery, S. Maria Misericordia Hospital, S. Andrea delle Fratte, 06132 Perugia, Italy; 6 Anaesthesia and Resuscitation Units, Franchini di Montecchio Emilia Hospital, 42121 Reggio Emilia, Italy; 7 Orthopaedics and Traumatology, Arcispedale S. Maria Nuova, 42121 Reggio Emilia, Italy

**Keywords:** hip fracture, mortality, NHFS, orthogeriatrics, functional performance

## Abstract

**Background/Objectives:** Older people with hip fractures (HF) are highly heterogeneous patients at risk of adverse events, which impacts healthcare and economic systems. Tools for estimating 30-day and 1-year mortality may help optimize patient management decisions. The Nottingham Hip Fracture Score (NHFS) is one of the most reliable tools for assessing 30-day mortality after surgery. The aim is to validate the NHFS in a cohort of Italian patients hospitalized due to HF and enhance its predictive capacity against 30-day and 1-year mortality. **Methods:** A prospective observational study among older patients with HF who underwent surgery after a comprehensive geriatric assessment (CGA). Data about 30-day and 1-year mortality were gathered from regional registries. Logistic regression analyses were performed to assess the predictive accuracy of the NHFS against 30-day and 1-year mortality. The adapted scores were measured using ROC curves, and a nomogram was developed. **Result:** Among 1169 patients, 30-day and 1-year mortality rates were 4.3% and 21.3%, respectively. The NHFS was validated and recalibrated in the study sample for 30-day mortality. The recalibrated NHFS showed better predictive capacity for 30-day mortality (AUC 0.693) than the ASA score (AUC 0.547) and remained a significant predictor for 1-year mortality (AUC 0.712). BADL and METs showed an association with mortality, so the modified NHFS was integrated, resulting in a more accurate prediction compared to the original score for 1-year mortality (AUC = 0.747). **Conclusions:** The NHFS is a reliable tool for estimating 30-day and 1-year mortality in Italian older adults with HF. The revised NHFS, which includes METs and BADL, offers higher predictive power than the original version in our sample, and the nomogram may facilitate its clinical use.

## 1. Introduction

Osteoporosis is a major public health concern, affecting one in three women and one in five men over the age of 50 [1,2]. Alongside falls, it is the primary driver of fragility fractures [3], a burden projected to rise to nearly six million cases annually by 2050 [4]. Despite reports of a decreasing trend among individuals who underwent total hip arthroplasty [5,6], hip fractures (HF) still represent a severe medical entity, imposing profound human, social, and economic costs [7].

Mortality following HF remains alarmingly high, with estimates of 29.17% at one year, 33.33% at two years, and 36.67% at three years post-fracture [8]. However, the disability burden associated with fragility fractures often exceeds mortality, particularly in individuals aged 50 to 85 years [9]. HF outcomes and patient management are significantly influenced by multisystemic complications, usually triggered by a combination of frailty and acute injury [10]. Key determinants of poor prognosis include advanced age [11], frailty status [12,13], pre-existing comorbidities [14,15], surgical technique [16,17], and timing of mobilization [18].

Optimizing perioperative risk stratification is crucial for enhancing individual outcomes and ensuring the sustainability of the healthcare system [19,20]. Several prognostic models have been developed to guide perioperative care, yet no single tool has demonstrated superiority in assessing perioperative risk among older adults. The American Society of Anesthesiologists (ASA) score remains the most widely used [21]. Alternative approaches, including frailty screenings and comprehensive geriatric assessment (CGA) [22], are frequently recommended to assist clinical decision-making for highly vulnerable HF patients [23,24]. The Nottingham Hip Fracture Score (NHFS) has emerged as a validated and clinically relevant risk assessment tool capable of predicting 30-day [25] and 1-year mortality in HF patients [26]. Based on admission data, including age, sex, malignancy, dementia, pre-operative living status, hemoglobin levels, predefined comorbidities, and frailty, inferred by identifying those living in long-term care facilities (LTCF), the NHFS provides a multidimensional, quantitative framework for early risk stratification in the pre-operative phase at the time of anesthesiologic assessment. The NHFS has been validated across different populations, including British, Dutch, Australian, Swedish, and Greek patients [27,28,29,30], as well as Asian [31,32] and Brazilian [33] older people.

Despite widespread international use, the NHFS remains unvalidated in the Italian healthcare context. Therefore, the primary aim of the study is to validate the NHFS within a cohort of Italian HF patients, explore any variables that could improve its predictive power, and then recalibrate the score based on participants’ features. The secondary endpoint, in case of low predictiveness of the NHFS, is to identify any variable capable of improving the prediction model in our sample. Implementing an Italian version of the NHFS can enhance care by identifying priorities, facilitating informed decision-making, improving communication with patients and families, and aligning treatment pathways with evidence-based best practices. Moreover, the validation of a preoperative risk assessment tool across different healthcare systems would enable meaningful data comparisons, fostering international collaboration and continuous improvement in HF management.

## 2. Materials and Methods

### 2.1. Study Design and Sample

**Figure 1:**
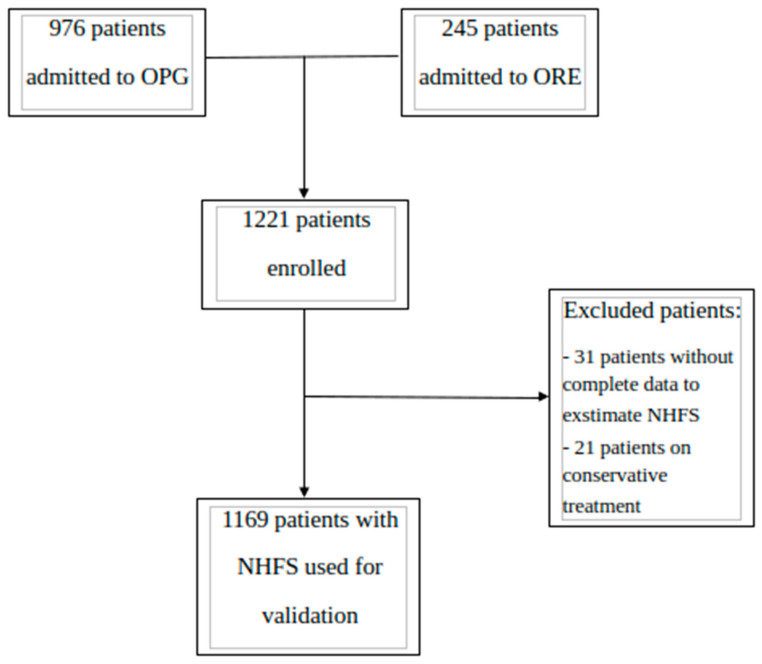
Flowchart of enrollment and inclusion processes according to STROBE. OPG: Perugia Hospital; ORE: Reggio Emilia Hospital.

Data were gathered from patients admitted to the Orthogeriatric Unit of Perugia (i.e., OPG) teaching hospital and the Orthogeriatric Unit of the Reggio Emilia (ORE) hospital. In accordance with the previous literature, patients were included in the study if they met the following criteria: (a) admitted due to low-energy HF [34,35], including femoral neck, intertrochanteric, subtrochanteric, and pathological fractures; (b) received surgical repair of HF by hip replacement or intramedullary nail fixation. As in the original study, no age limit was set to expand the probability of including patients with the lower bands of NHFS. Subjects were not included if they had suspected non-osteoporotic fracture, were receiving terminal palliative care, or refused surgery; in addition, participants were excluded if they died before surgery or had missing data regarding variables needed to calculate the NHFS, as shown in Figure 1.

Based on the available resources at participating units, patients were identified among those consecutively admitted in specific time windows from October 2018 to September 2023, with most of them enrolled from January to December 2021 at OPG (n. 519, 44.4%) and from January to June 2021 at ORE (n. 245, 20.9%).

After excluding 11 patients due to incomplete information about items needed for NHFS estimation, the final sample consisted of 1169 HF patients (Figure 1). The manuscript was written in accordance with the STROBE checklist.

The study was consistent with the Helsinki Declaration’s ethical standards. The regional healthcare system ethics committee approved the study with registration number 2257/14 at OPG and with registration number 1465 (EMAFEM_GERI15_22) at ORE.

### 2.2. Data Variables and Sources

#### 2.2.1. NHFS

According to the NHFS, the 30-day mortality risk associated with HF surgery is based on the following variables and related points: age (≤65 years old, 0 points; 66–85 years old, 3 points; ≥86 years old, 4 points), gender (male, 1 point; female, 0 points), hemoglobin at admission (≤10 g/dL, 1 point; ≥10 g/dL, 0 points), presence of any active malignant tumors within 20 years (yes, 1 point; no, 0 points), the Abbreviated Mental Test Score (AMTS) to detect cognitive dysfunction at admission (a cutoff ≤6 indicates moderate–severe cognitive dysfunction associated with dementia), and the number of comorbidities (≥2 comorbidities, 1 point; ≤2 comorbidities, 0 points) based on a predefined list as reported by Maxwel et al., 2008 [25], including myocardial infarction or angina, atrial fibrillation, valvular heart disease, hypertension, stroke, COPD or asthma, and chronic kidney disease. Points were assigned as per the original studies, except for dementia, since the Abbreviated Mental Test Score (AMTS) was not routinely performed. In the original NHFS, patients’ performance on the AMTS was used to establish whether dementia was present. In our study, any clinical note documenting dementia or cognitive impairment was used as a surrogate variable for this test, as previously described by Nijmeijer et al., 2016 [36]. If patients presented with “loss of awareness” and “confusion” during clinical exams, we inferred an AMTS of ≤6, which is the cutoff for cognitive dysfunction in the NHFS. On the other hand, if presented with “awareness” or “oriented in space and time,” we considered AMTS > 6. The participant was excluded if there were conflicting opinions or if it was impossible to obtain information, as in Ferro et al., 2025 [33]. Data needed to estimate NHFS and to describe patients’ characteristics were collected from medical charts through a detailed inquiry, especially concerning cardiovascular diseases (i.e., myocardial infarction, angina pectoris, atrial fibrillation, valvular disease, and hypertension), cerebrovascular diseases (i.e., having experienced a stroke or ischemic diseases), chronic respiratory and kidney diseases (i.e., except acute infection and kidney injury) [25], and diabetes. A standard colorimetric assay measured hemoglobin. The NHFS was calculated by combining the points attributed to the conditions mentioned above. Since the original formula for the conversion of NHFS to prognosticate 30-day mortality has been revised, the 2012 iteration of the formula was used for validation in the present study [37]. The actual NHFS mortality rates, available online, were checked for comparisons [38].

#### 2.2.2. Patients’ Characteristics and Variables

All patients underwent assessments of perioperative risks using the ASA score [21], which is mandatory and reported in the clinical chart, together with the Metabolic Equivalent of Tasks (METs) [39]. The ASA spans from I (healthy) to VI (brain dead), according to the anesthetist’s evaluation, as previously described [21]. According to the Older Adult Compendium of Physical Activities, patients had a MET score ≥ 4 if they were at least able to walk and climb stairs, then received 1 point, while those with poor functional capacity, i.e., unable to walk and climb stairs, had a score < 4 and received 0 points [40].

A CGA was performed at hospital admission, allowing for the collection of information about pre-fracture functional status and mobility, caregiver needs, living arrangements, comorbidities, and medications, which were recorded by class and quantity. Pre-fracture functional status was assessed using the Basic Activities of Daily Living (BADL) and Instrumental Activities of Daily Living (IADL). Independence was defined as an IADL score of >6 points for women and >3 for men, and an ADL score of >5 for both genders. For BADL, a score ≥ 5 was assigned 1 point, and a score ≤ 4 received 0 points [41]. Living dependency was evaluated based on the place of living declared at admission; long-term care settings and home care were included. According to the American Orthopedic Society, the fractures were categorized as medial, i.e., subcapital, and basicervical, lateral, i.e., pertrochanteric, and subtrochanteric, then distal as diaphyseal [42]. All data were collected and coded by medical doctors. All patients were checked against the national death registry for 30-day and 1-year mortality dates.

#### 2.2.3. Statistical Analyses

The Shapiro–Wilk test was used to assess the normal distribution of variables. The Chi-square test with Yates’ continuity correction and Fisher’s exact test were used for comparisons of categorical variables, and the Mann–Whitney U-test was used for comparisons of ordinal and non-normally distributed continuous variables. To examine the risk factors affecting the prognosis, bivariate and multivariate logistic regression models were fitted, incorporating in the multivariate model as explanatory variables all the variables that showed a *p*-value < 0.1 in bivariate analysis. To avoid the multicollinearity problem, highly correlated variables were dropped from the models. To decrease the overfit bias and internally validate our results, all bivariate and multivariate regressions were subjected to 200 bootstrap resamples, and their goodness-of-fit was tested using the Hosmer and Lemeshow test and calibration plot. Odds ratios (ORs) with 95% confidence intervals were also calculated. Furthermore, multivariate logistic regression coefficients were used to develop a risk factors-based nomogram, and its predictive accuracy was internally validated using the Area Under the Receiver Operating Characteristic curve (AUC), built on the probability of having the outcome derived from logistic regression models, and the AUCs were compared by the DeLong method. Statistical analysis was performed using IBM-SPSS^®^ version 26.0 (IBM Corp., Armonk, NY, USA, 2019). In all analyses, a two-sided *p*-value < 0.05 was considered significant. Nomogram and calibration plots were developed using the NOMOLOG [43] and PMCALPLOT [44] Stata modules, respectively (StataCorp, 2015; Stata Statistical Software, Release 14; College Station, TX, USA: StataCorp LP). To evaluate the accuracy of the predictive models, we also used decision curve analysis (DCA) [45]. A decision curve (DC) plots net benefit for a given model across a range of threshold probabilities. Net benefit is calculated as true positives minus false positives (both expressed as a fraction of the total number of subjects), with the false-positive term weighted by the threshold probability.

$$\mathrm{NetBenefit}=\frac{\mathrm{True}\;\mathrm{Positives}}{n}-\left(\frac{\mathrm{False}\;\mathrm{Positives}}{n}\cdot \frac{p_{t}}{\left(1-p_{t}\right)}\right)$$

Assuming that the probability thresholds are the same and fixed between the decision curves, the frequencies of both true and false positives in each probability class remain the only determinants of different curve shapes. Therefore, to compare the decision curves, the goodness-of-fit X2 test has been applied, considering the expected frequencies to be those on which we built the first DC, and observed frequencies to be those on which we built the second DC, or vice versa. In a goodness-of-fit X2 test, not equal and/or non-integer expected frequencies are also allowed, but we have to be sure that (1) the marginal total is the same for both the observed and expected frequencies, (2) there are no expected frequencies less than 1, and (3) no more than 20% of the expected frequencies are less than 5. Decision curves were plotted using the DCA Stata module (StataCorp, 2015; Stata Statistical Software, Release 14; College Station, TX, USA: StataCorp LP). In the Supplementary Materials, Figure S4 presents the decision curve analysis for the models. In both DCAs, the assumptions of the goodness-of-fit X2 test were not violated across a range of probability thresholds from 5% to 80%, and the decision curve shapes were significantly different between the model with NHSF alone and the final model (*p* < 0.0001), showing a greater net benefit of the latter along the entire curve.

## 3. Results

### 3.1. Participants’ Characteristics According to NFHS

Table 1 reports the characteristics of the 1169 participants who underwent surgery to repair HF in the entire sample and according to 30-day vital status. On average, patients were 83.4 ± 8.3 years old, and the higher the age, the higher the mortality rate. The 30-day mortality rate was 4.3% (*n* = 51), with no events in patients younger than 65 years; 20 deaths (39.2%) occurred in those aged 65–85, and 31 (60.8%) in those older than 85 years. Overall, the average NHFS was 4.7 ± 1.5, with a higher score among those who died than those who were still alive after 30 days from surgery (5.7 ± 1.3 vs. 4.7 ± 1.5; *p* < 0.0001). Women (*n*: 877; 75%) were more prevalent than men, especially in the 65–85 year age range (*n*: 610; 52.2%), and were more likely to survive 30 days after surgery (75.8% vs. 56.9%; *p* 0.004) compared to men. Hemoglobin at admission was 12 gr/dL on average, with lower levels among those with 30-day mortality compared to those who survived (11.1 ± 1.8 vs. 12.1 ± 1.7; *p* 0.001). One out of three patients had dementia (*n*: 394, 33.7%), and the prevalence of survivors was higher among those with mild compared to moderate anemia (84.7% vs. 31.4%; *p* 0.004). Only 4.6% (*n*: 54) were long-term residents, and did not show a difference in their 30-day vital status. Overall, malignancy in the last 20 years was reported in 18.4% (*n*: 215) of patients, with a similar distribution (19.6% vs. 18.3%, *p* 0.965) between decedents and survivors. About 42.1% (*n*: 492) of participants had two or more comorbidities, with higher prevalence in those who died compared to those who survived (64.7% vs. 41.1%, *p* 0.001). Hypertension (71.3%) was the most prevalent disease, followed by atrial fibrillation (17%), which was highly prevalent among decedents (*p* 0.003), then COPD and asthma (15.7%), chronic kidney disease (13.9%), myocardial infarction or angina (11.5%), stroke (9.2%), and valvular heart disease (8.6%). Notably, approximately 63% (*n*: 735) of patients were taking 5 or more medications, and 14% (*n*: 164) were on 10 or more drugs, and did not show differences in 30-day vital status. Lateral (52.9%) hip fractures were more prevalent than medial (43.1%) fractures in the entire sample, with similar distribution between 30-day survivors and decedents. Most patients (*n*: 988; 84.5%) had an ASA score of class 3, and few reported class 2 (*n*: 90; 7.7%) and class 4 (*n*: 91; 7.8%). Overall, 80% of decedents and 85.1% of survivors scored 3 on the ASA (*p* 0.179). About half (*n*: 568; 48.6%) of the patients were independent in daily functioning before fracture, and 39.5% (*n*: 461) reported METs equal or higher than 4, indicating their ability to climb stairs. Pre-fracture BADL independence was highly prevalent among those still alive 30 days from surgery compared to those decedents (50.0% vs. 17.8%; *p* < 0.0001). Similar trends were found about pre-fracture IADL independence both in men and women.

### 3.2. NHFS and 30-Day Mortality

Table 2 presents the observed mortality rates across NHFS risk classes, alongside the mortality predicted by the original NHFS models and actual NHFS mortality rates, available online [38]. From 0 to 10 NHFS risk classes, the majority of patients scored 5 (*n*: 311; 27.8%), followed by 4 (*n*: 269; 24.1%), 6 (*n*: 214; 19.1%), 3 (*n*: 177; 15.8%), and 7 (*n*: 88; 7.9%). Consistently, patients who experienced 30-day mortality mainly belonged to NHFS risk class 6 (31.4%), followed by risk classes 5 (23.5%), 7 (21.6%), 4 (11.8%), 3 (5.9%), and 8 (3.9%). Mortality increased progressively with higher NHFS, ranging from 0.3% in NHFS category 0 to 34.5% in category 10. The mortality trends associated with higher NHFS risk classes in our cohorts were similar to those reported from previous studies by Maxwell et al., 2008 [25], Olsen et al., 2021 [29], and Moppett et al., 2012 [37] (Table 2).

The predicted mortality per each class risk, estimated using logistic regression, was lower in our cohort compared to those previously reported from the above-mentioned studies, suggesting a progressive decline in mortality, with current rates being lower than those reported in 2008 and 2012. As noted in Table 2, the logistic regression analysis provided updated coefficients for the NHFS formula with *P* represented as the predicted mortality percentage:

$$P\left(\%\right)\;=\;\frac{100}{1\;+\;e\left(5.75-\left(0.511\;\times \;\mathrm{NHFS}\right)\right)}$$

The recalibrated model’s accuracy was validated using the Hosmer and Lemeshow test and calibration plot (Supplementary Figure S1), showing that the expected and observed frequencies have a fairly good correspondence. Interestingly, the predicted mortality in our cohort does not substantially differ from the current UK-based risk estimates [38]. Finally, the predictive capacity of actual values of NHFS versus ASA score for 30-day mortality was tested, showing significant difference (*p*-value = 0.0003, Supplementary Figure S2).

### 3.3. NHFS and 1-Year Mortality

Based on previous evidence [26,28,32] and the considerations outlined above, we therefore considered it appropriate to validate the NHFS to one-year mortality in an Italian cohort. Table 3 presents the main predictors of 1-year mortality in our sample, as determined by multivariate logistic regression models.

**Figure 2:**
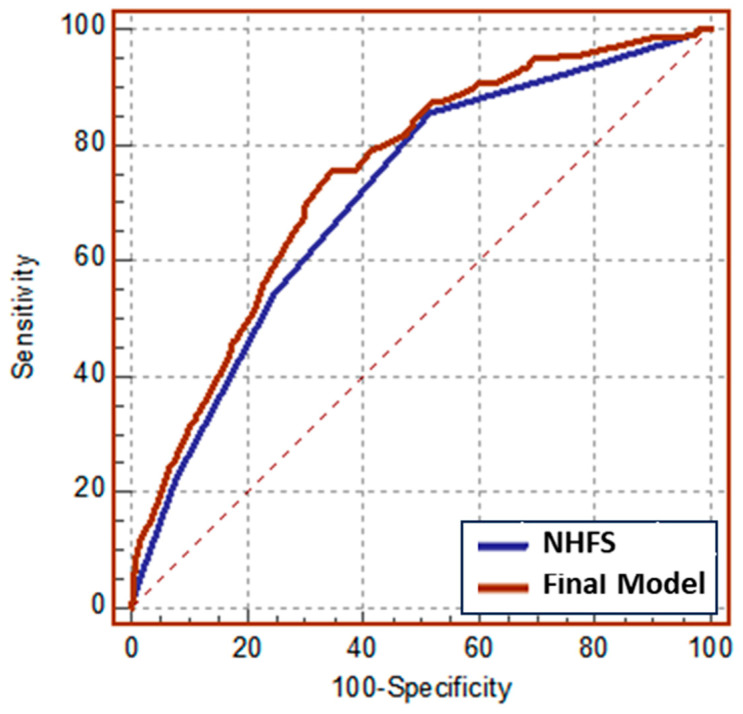
ROC curves of logistic models for 1-year mortality among orthogeriatric patients.

Multivariate model confirmed NHFS as a statistically significant predictor of 1-year mortality, independent of medications, ASA, BADL, and METs. Since the last two variables showed a *p* < 0.1 towards 1-year mortality, we refined the prediction model for 1-year mortality reported as the Final model, showing a higher predictive power compared to the other variables analyzed (Table 3, Final model). Then, we formally tested the predictive capacity of NHFS alone versus the Final model by comparing the AUC built on predicted probabilities of being a case or control derived from logistic regression. As presented in Figure 2, there was a statistically significant difference (*p* 0.0056) between the AUC NHSF alone, which equals 0.712, and the AUC Final model, which equals 0.747 (Table 4).

**Figure 3:**
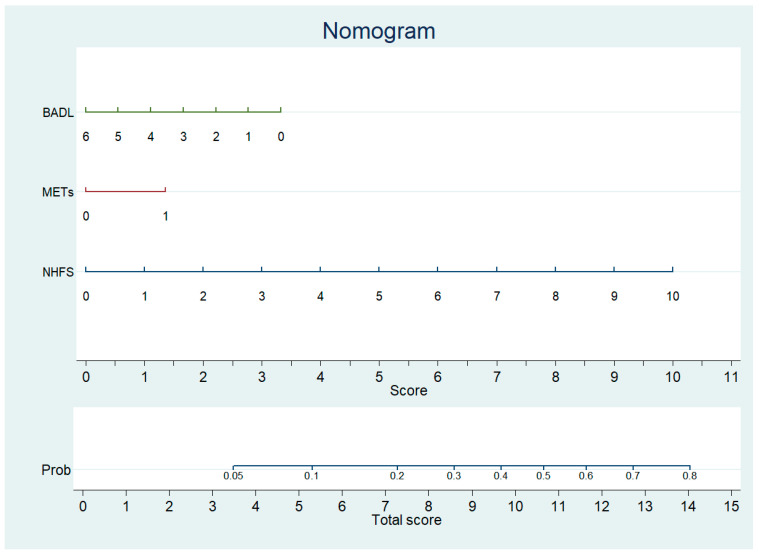

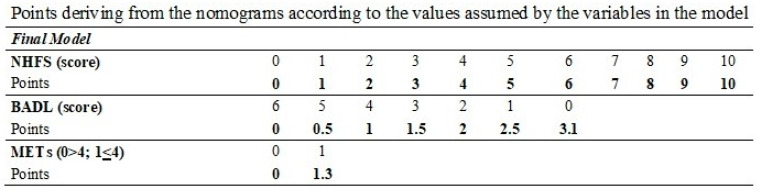
Nomogram predicting 1 year mortality after surgery to repair HF based on NHFS, METs, and BADL.

Therefore, we concluded that the NHFS, combined with the assessment tools measuring the level of functional autonomy (BADL + METs), may improve the prediction of 1-year mortality in our sample. A nomogram for predicting 1-year mortality was developed using estimates from the logistic model (Table 3). The nomogram for predicting mortality likelihood at hospital admission includes three variables: NHFS, pre-fracture BADL, and METs. The nomogram calculates a score by summing the points corresponding to each predictor variable. The probability of 1-year mortality at hospital admission (Figure 3) was derived from the total score.

Calibration plots for internal validation of the model are reported in the Supplementary Materials (Supplementary Figure S3). The plots showed a good agreement between the estimated and the actual 1-year mortality frequencies, with an AUC of 0.747. The table at the bottom of Figure 3 reports the points to be used according to the values assumed by the variables in the nomogram.

Furthermore, the logistic regression model provided the coefficients to solve the new equation for the 1-year mortality, where P represents the predicted death percentage:

$$P\left(\%\right)\;=\;\frac{100}{1\;+\;e\left(3.045-\left(0.429\;\times \;\mathrm{NHFS}-0.218\;\times \;\mathrm{BADL}\;+\;0.573\;\times \;\mathrm{METs}\right)\right)}$$

## 4. Discussion

This is the first study validating the NHFS against 30-day and 1-year mortality among a multicentric cohort of Italian older adults consecutively admitted to hospital due to low-trauma HF and who underwent surgical repair. An optimized version of the NHFS, which includes measures of pre-fracture functioning, was tested and proposed to enhance mortality estimations and support professionals’ decision-making.

In our sample, the NHFS demonstrated a prediction capacity for 30-day mortality, with a similar trend but lower absolute rates for each score category, compared to those reported in previous studies [25,37]. Furthermore, the absolute capacity to predict 30-day mortality after surgery appears to be more accurate than that estimated by the ASA score. Interestingly, the expected mortality in our cohort does not substantially differ from that observed in the current UK-based risk estimates [38].

Differences in the 30-day and 1-year mortality rates due to changes in the overall longitudinal trend may help to explain discrepancies: in our sample, 30-day mortality is 4.3% in 2020 compared to 7.8% reported by Maxwell’s study in 2008, and 6.6% by Moppet in 2012 [37], but similarly to what is currently observed in the National Healthcare System in UK [38]. We can argue that the absolute capacity to predict 30-day mortality might be affected by the optimization and spreading of orthogeriatric competencies and best practices. The recalibrated version of the score led to an Italian version taking into account the population’s features, orthogeriatric co-management with early CGA as a milestone, and early surgery being recommended and nationally monitored [46]. Our hypothesis is consistent with findings of studies conducted in other countries. For instance, the mortality rates remain higher than those reported in our study, where early surgery is not yet considered the gold standard for treatment [33], orthogeriatric services are not in place [30], or mortality rates did not take into consideration patients receiving conservative treatments of their HF [27]. In Italy, the knowledge and interest about the orthogeriatric care model is increasingly widespread, with several hospitals across the country working together continuously to promote best practices [46,47].

One-year mortality after surgical repair of HF was 21.3% in our sample, slightly lower than the 29.17% found in Turkey [8], and 29.3% in England [26], and almost superimposed to that summarized at the EU level by Medin et al., 2015 [48]. However, it was higher than the 14.6% reported by Kau et al., 2014 in a small sample of older adults aged 60 years and more, surgically treated from September 2009 to April 2010 [32]. Then, this is the first study showing the predictive capacity of NHFS for 1-year mortality in an Italian sample of HF patients. The mortality rates observed in the Italian version of the NHFS demonstrated a proportional trend with increasing NHFS risk classes. An NHFS > 4 was reported in 96% of patients who died within 30 days and in 94% of those who died within 1 year after surgery, rising in step with older age and comorbidities, more steeply in men and in anemic patients.

We also showed that cardiovascular (in particular atrial fibrillation), respiratory, and kidney diseases proved to be of greater importance, and unlike what was found by Ferro et al, 2025 [33], diabetes did not show a significant correlation with 30-day mortality in our sample. Similarly to Camur et al., 2019, polypharmacy confirmed an association with 1-year mortality [49], but previous neoplastic disease or institutionalization did not show a significant correlation with 30-day mortality. As already shown by Castronovo et al., 2011, in an Italian sample, we confirmed the negative impact on 1- and 6-month mortality of age, gender, comorbidities, and cognitive dysfunction [50]. Additionally, pre-fracture BADL and METs values resulted in independent predictors for both 30-day and 1-year mortality [51]. Building on the predominant role of the pre-fracture functional performance and mobility compared to usual risk factors, we tested and confirmed the better predictive capabilities of the score after testing BADL and METs, as previously valued by Stubs et al., 2023 [52].

METs have never been studied before in the orthogeriatric setting. However, it is recommended for cardiac risk stratification before non-cardiac surgery [53]. METs proved to be a functional and cost-effective parameter that can estimate individual physical fitness and overall physiological reserve [40]. Therefore, an optimized version of the NHFS is proposed to better adapt to the Italian population, as already performed by the Greek [30] and Dutch researchers [36], who also included pre-fracture mobility parameters to optimize their tools. These efforts underscore the importance of considering multiple domains when assessing prognosis in frail older patients, adding insight into the complexity of predicting mortality and offering more appropriate tools for daily practice [54]. The need for tools supporting decision-making is a national willingness and sensitive goal, as recently shown by the Fondazione Policlinico Gemelli (FPG) score, which proposes risk stratification at the Emergency Department (ED) [55]. Consequently, we developed a nomogram for an easy implementation from the ED by multiprofessionals with minimum orthogeriatric competencies.

The availability of the NHFS validated for the Italian population offers, for the first time, the opportunity to better identify HF subjects at high risk for mortality. The NHFS enhanced through the integration of multidimensional and pre-fracture mobility parameters further improves the identification of high-risk patients in the short (30 days) and long term (one year). On one side, the enhanced NHFS may be a valuable tool for guiding professionals, patients, and caregivers through the difficult choice between surgical and conservative treatment in the short term. On the other side, the 1-year prediction may help the decision-making beyond the surgical step, focusing on the best care and practices over the entire pathway, ultimately offering personalized treatment to improve quality of life.

We acknowledge some limitations of the study. First, the exclusion of patients receiving conservative treatment could represent a lack of information. However, recommendations suggest that less than 4% receive conservative treatment, and these are mostly terminally ill patients, who require different evaluations and goals than those just focused on time to mortality. Second, we acknowledge the potential bias due to the lack of AMTS use; however, as a pragmatic solution, we identified patients with dementia or cognitive impairment by clinical comprehensive assessment and data mining from the clinical charts made by expert geriatricians, as did other authors [27,36]. Third, the limited number of events observed within 30 days since surgery can be considered a limitation. Indeed, according to some authors, a number of events less than 100 may bring poor intrinsic validity for a validation study. However, many studies do not follow this rule [56], and we calculated that the sample size has sufficient statistical power for our country in consideration of the incidence of mortality observed in the study group (4.3%) and the one registered for the Italian population in 2021 (6.42%) from PNE 2023 [46,57]. Since a number of events might result in a limited prediction capability in the short term, e.g., 30 days, extending the estimation to the long term, e.g. 1 year, may improve statistical predictivity, especially if other variables are associated with the NHFS, as in our study.

## 5. Conclusions

We propose an Italian validated version of the NHFS, taking into account the most advanced standard of care in two regional healthcare areas. The recalibrated NHFS version appears to be a reliable tool for estimating 30-day and 1-year mortality from HF surgical repair.

Moreover, the inclusion of pre-fracture functional status may improve risk stratification among orthogeriatric patients, as already shown in models for cardiac surgery [54]. A nomogram based on the Italian optimized version of the NHFS was developed. The modified Italian NHFS seems a more appropriate risk stratification tool for orthogeriatric setting compared with ASA and the original version, however additional studies are required to confirm these findings before widespread use.

## Figures and Tables

**Table 1:** Patients’ characteristics in the entire sample and according to the 30-day post-surgical vital status.

NHFS Related Variables	Entire Sample at Admission (n. 1169; 100%)	Decedent 30-Day Post-Surgery (n. 51; 4.3%)	Alive 30-Day Post-Surgery (n. 1118; 95%)	*p* Value
NHF score, mean + SD	4–7 ± 1.5	5.7 ± 1.3	4.7 ± 1.5	<0.0001
Women, n (%)	877 (75.0)	29 (56.9)	848 (75.8)	0.004
Age, years	83.4 ± 8.3	87.3 ± 6.6	83.2 ± 8.4	0.001
Age class, n (%)				
years < 64 years	36 (3.1)	0 (0)	36 (3.2)	0.04
65 < years < 85	610 (52.2)	20 (39.2)	590 (52.8)	
>85 years	523 (44.7)	31 (60.8)	492 (44)	
Haemoglobin at admission, gr/dl	12.0 ± 1.7	11.1 ± 1.8	12.1 ± 1.7	0.001
Anemia classes, n (%)				
Moderate (Hb <10 g/dL)	187(16)	16 (31.4)	171 (15.3)	0.004
Mild (Hb > 10 g/dL	982 (80)	35 (68.6)	947 (84.7)	
Dementia	394 (33.7)	24 (47.1)	372 (32.1)	0.056
Institutionalization	54 (4.6)	2 (3.9)	52 (4.6)	0.712
Malignancy within last 20 years	215 (18.4)	10 (19.6)	205 (18.3)	0.965
Specific comorbidities, >2	492 (42.1)	33 (64.7)	459 (41.1)	0.001
Myocardial infarction or angina	135 (11.5)	11 (21.6)	124 (11.1)	0.039
Atrial fibrillation	199 (17)	17 (33.3)	182 (16.3)	0.003
Valvular heart disease	100 (8.6)	7 (13.7)	93 (8.3)	0.274
Hypertension	834 (71.3)	37 (72.5)	797 (71.3)	0.971
Stroke	108 (9.2)	8 (15.7)	100 (8.9)	0.168
COPD or asthma	184 (15.7)	14 (27.5)	170 (15.2)	0.031
Chronic kidney disease	163 (13.9)	11 (21.6)	152 (13.6)	0.161
**Additional patients’ characteristics**				
Type of fracture				
Medial	504 (43.1)	23 (45.1)	481 (43)	0.72
Lateral	618 (52.9)	25 (49)	593 (53)	
Distal	47 (4)	3 (5.9)	44 (4)	
ASA score, n (%)				
II	90 (7.7)	2 (3.9)	28 (7.9)	0.179
III	988 (84.5)	42 (82.4)	946 (84.6)	
IV	91 (7.8)	7 (13.7)	84 (7.5)	
METs	Media + SD	Media + SD	Media + SD	
METs < 4	708 (60.6)	48 (93.2)	660 (59.1)	<0.0001
ADL	4.0 ± 1.8	2.9 ± 1.5	4.0 ± 1.7	
ADL > 5	513 (48.6)	8 (17.8)	505 (50)	
IADL				
Women > 6	265 (30.3)	2 (7.7)	264 (31.1)	0.001
Men > 3	157 (53.9)	5 (21.1)	153 (56.5)	0.004
Medication at admission, numbers	5.9 ± 3.2	6.7 ± 3.1	5.8 ± 3.2	0.064
>5	753 (62.9)	35 (68.4)	701 (62.7)	0.478
>10	164 (14)	9 (18.4)	153 (13.8)	0.576

Data are presented as mean and SD, and proportions and percentages as appropriate, otherwise as reported.

**Table 2:** Distribution of the cohort by classes and size of the NHFS categories, and of the predicted mortality at 30 days based on internally revised NHFS in comparison with the predicted mortality from the studies originated by Maxwell et al., 2008 [25]; Moppet et al., 2012 recalibration [37], and the Swedish validation by Olsen et al., 2021 [29].

Study Cohort				Comparison Studies			
NHFS Score Risk Classes	Total Study Cohort n. (%)	Mortality Rate Observed n. (%)	Mortality Rate Predicted According to Revised NHFS	Mortality Rate Predicted According NHFS			Actual NHFS Online Rate *
				Maxwell [25]	Moppet [37]	Olsen [29]	
0	19 (1.7)	0 (0)	0.3	0.9	0.7	1.7	0.4
1	15 (1.3)	0 (0)	0.5	1.5	1.1	2.5	0.6
2	4 (0.4)	0 (0)	0.9	2.4	1.7	3.7	1
3	177 (15.8)	3 (5.9)	1.5	3.8	2.7	5.4	1.7
4	269 (24.1)	6 (11.8)	2.4	6.2	4.4	7.8	2.8
5	311 (27.8)	12 (23.5)	3.9	9.8	6.9	11.2	4.6
6	214 (19.1)	16 (31.4)	6.4	15.2	10.7	15.8	7.4
7	88 (7.9)	11 (21.6)	10.2	22.8	16.2	21.9	11.8
8	19 (1.7)	2 (3.9)	15.9	32.8	23.8	29.5	18.2
9	1 (0.1)	1 (2)	24	44.6	33.6	38.5	27
10	1 (0.1)	0 (0)	34.5	57	45	48.3	38

* Actual mortality rates were estimated for each NHFS category using the online calculator available at www.thereisafracture.co.uk/NHFS/index.htm (accessed on 4 November 2025).

**Table 3:** Predictors of 1-year mortality among HF patients using multivariate logistic regression models.

*Multivariate model*			
	OR (95% CI)	*p*-Value	B (SE)
NHFS	1.492 (1.292–1.722)	<0.0001	0.400 (0.07)
BADL score	0.797(0.707–0.898)	<0.0001	−0.227 (0.06)
METs < 4	1.589 (0.967–2.613)	0.068	0.463 (0.25)
ASA score	1.261 (0.796–1.998)	0.324	0.232 (0.23)
Medication at admission, *n*.	1.008 (0.955–1.064)	0.773	0.008 (0.03)
Final model *			
	**OR (95% CI)**	***p*** **-value**	**B (SE)**
NHFS	1.535 (1.340–1.759)	<0.0001	0.429 (0.07)
BADL score	0.804 (0.716–0.903)	<0.0001	−0.218 (0.06)
METs < 4	1.773 (1.088–2.891)	0.022	0.573 (0.25)

*OR* Odds ratio, *CI* confidence interval, *B* regression coefficient, *SE* standard error. * All variables with *p* < 0.1 in the initial multivariate analysis were included in a subsequent backward selection and elimination process until the final model was obtained.

**Table 4:** Comparison between NHFS and final model in the logistic regression.

	AUC	SE ^a^	95% CI ^b^
**NHFS**	0.712	0.0188	0.682 to 0.740
**Final Model**	0.747	0.0182	0.719 to 0.774
**Pairwise comparison of ROC curves**			
**NHSF versus Final Model**			
Difference between areas			0.0351
Standard Error ^c^			0.0127
95% Confidence Interval			0.0103 to 0.0599
z statistic			2.771
Significance level			*p* = 0.0056

^a^ *SE* standard error, ^b^ *CI* confidence interval; ^c^ DeLong et al., 1988.

## Data Availability

The data presented in this study are available on request from the corresponding author.

## Supplementary Materials

The following supporting information can be downloaded at: https://www.mdpi.com/article/10.3390/jcm15010310/s1, Figure S1: Calibration plot of predictive capacity of NHFS for 30-day mortality; Figure S2: Comparison of ROC curves on 30-day mortality risk prediction. NHFS (blu line—AUC: 0.693); ASA score (red line—AUC: 0.547); Figure S3: Calibration plot of predictive capacity of Final model with NHFS + BADL + METs for 1 year mortality; Figure S4: Decision curve analysis for the models shown in Figure 2.
