# Supplementary material for: Validation and Adaptation of the Nottingham Hip Fracture Score to Predict 30-Day and 1-Year Mortality Among Italian Older Adults Hospitalized Due to Hip Fractures
Source: J Clin Med. 2025 Dec 31;15(1):310. doi: 10.3390/jcm15010310 (PMC12787008; doi:10.3390/jcm15010310)
Supplement: Supplementary file 1 [file jcm-15-00310-s001.zip › jcm-4000119-supplementary.pdf]

Supplementary Materials

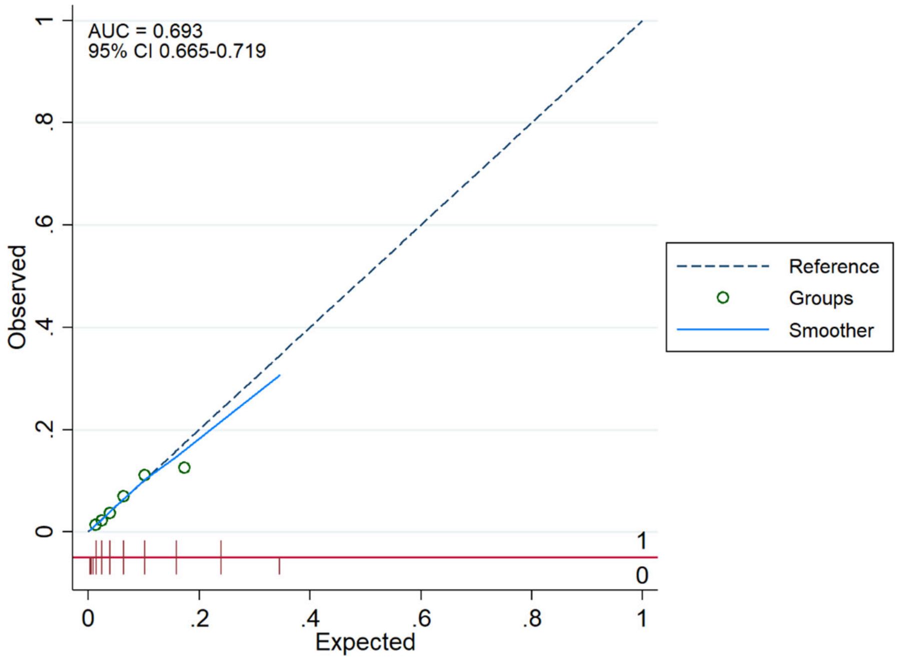

Figure S1. Calibration plot of predictive capacity of NHFS for 30-day mortality.

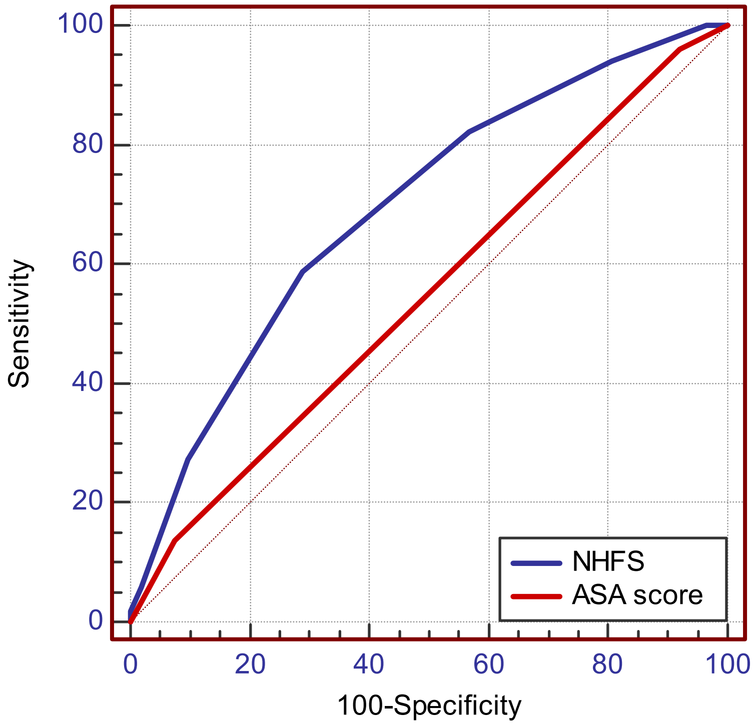

Figure S2. Comparison of ROC curves on 30-day mortality risk prediction. NHFS (blu line- AUC: 0,693); ASA score (red line– AUC: 0,547).

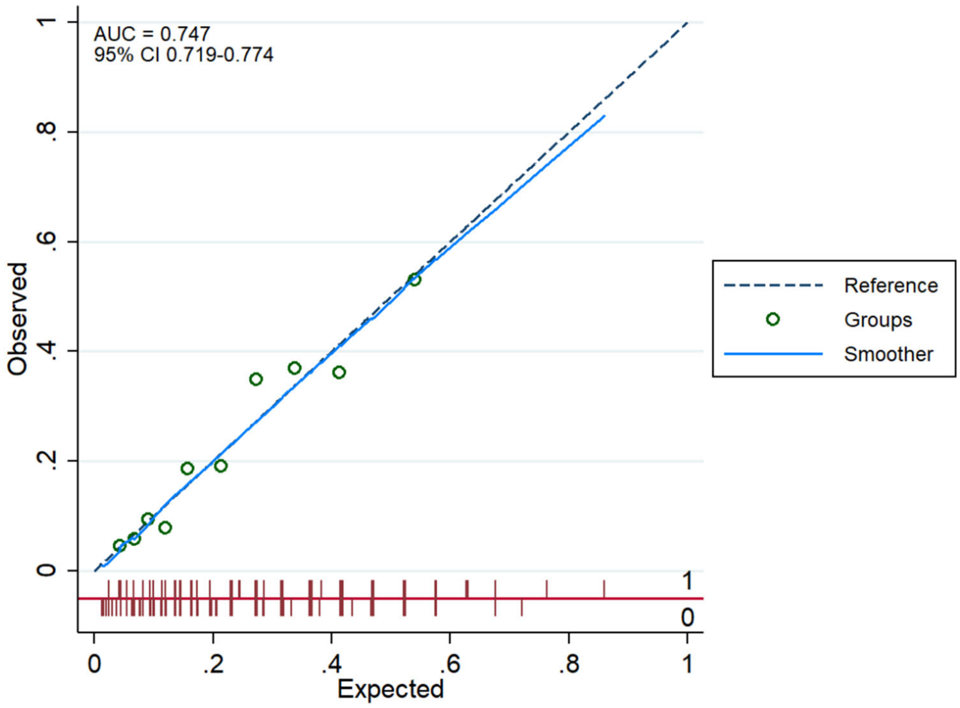

Figure S3. Calibration plot of predictive capacity of Final model with NHFS + BADL + METs for 1 year mortality.

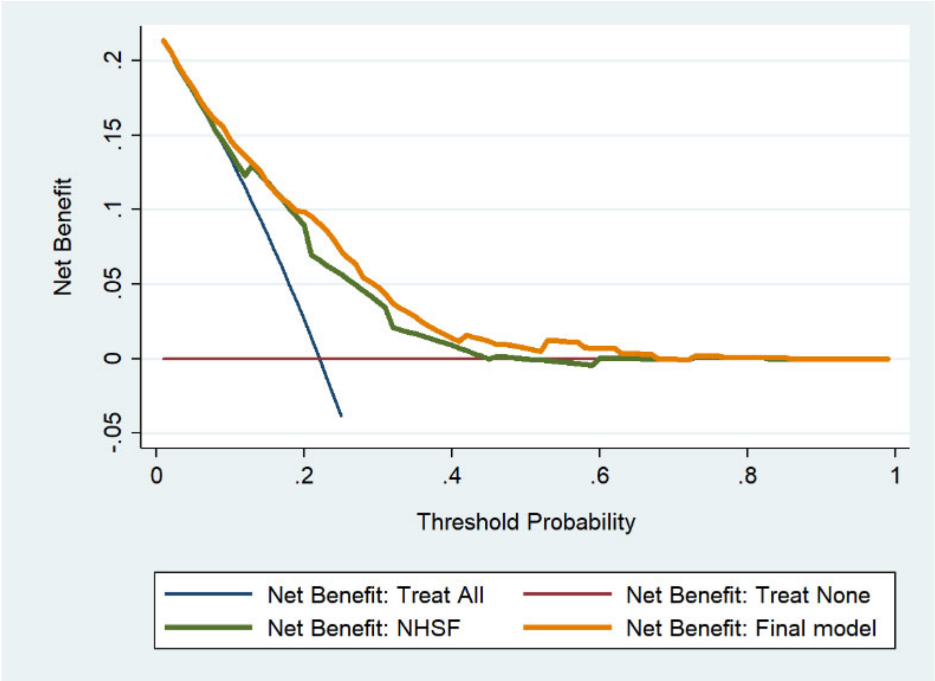

Figure S4. Decision curve analysis for the models showed in Figure 2.
